# Supplementary material for: Novel Thermostable Heparinase Based on the Genome of Bacteroides Isolated from Human Gut Microbiota
Source: Foods. 2022 May 18;11(10):1462. doi: 10.3390/foods11101462 (PMC9141863; doi:10.3390/foods11101462)
Supplement: Supplementary file 1 [file foods-11-01462-s001.zip › Supplementary data.pdf]

Supplementary data

Table S1. Analysis of the CAZy database PL13 family and the Centrum bacteriophage library.

Number	Organism	Database	Speciality
1	*Bacteroides caecimuris*	CAZy	
2	*Bacteroides cellulosilyticus*		
3	*Bacteroides helcogenes*		
4	*Bacteroides heparinolyticus*		
5	*Bacteroides intestinalis*		
6	*Bacteroides ovatus*		
7	*Bacteroides sp*		
8	*Bacteroides stercoris*		
9	*Bacteroides thetaiotaomicron*		
10	*Bacteroides xylanisolvens*		
11	*Bacteroides uniformis*	Our Center	Y
12	*Bacteroides fragilis*		Y
13	*Bacteroides thetaiotaomicron*		
14	*Bacteroides caccae*		Y
15	*Bacteroides eggerthii*		Y
16	*Bacteroides merdae*		Y
17	*Bacteroides cellulosilyticus*		
18	*Bacteroides ovatus*		
19	*Bacteroides clarus*		Y
20	*Parabacteroides distasonis*		Y
21	*Bacteroides vulgtus*		Y
22	*Bacteroides stercoris*		
23	*Bacteroides nordii*		Y
24	*Bacteroides finegoldii*		Y
25	*Bacteroides cellulosilyticus*		
26	*Bacteroides xylanisolvens*		
27	*Bacteroides sp*		

Y means only present in Our Center.

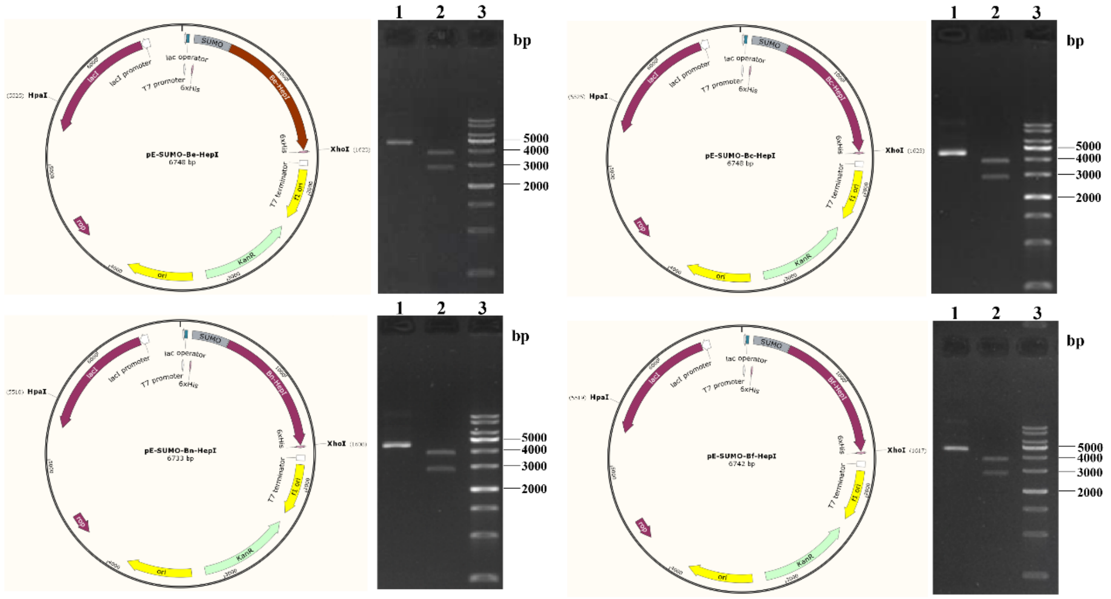

Figure S1. The heterologous expression vector was constructed with the fusion tag SUMO-Tag and verified using double digestion with HpaI and XhoI.

a b

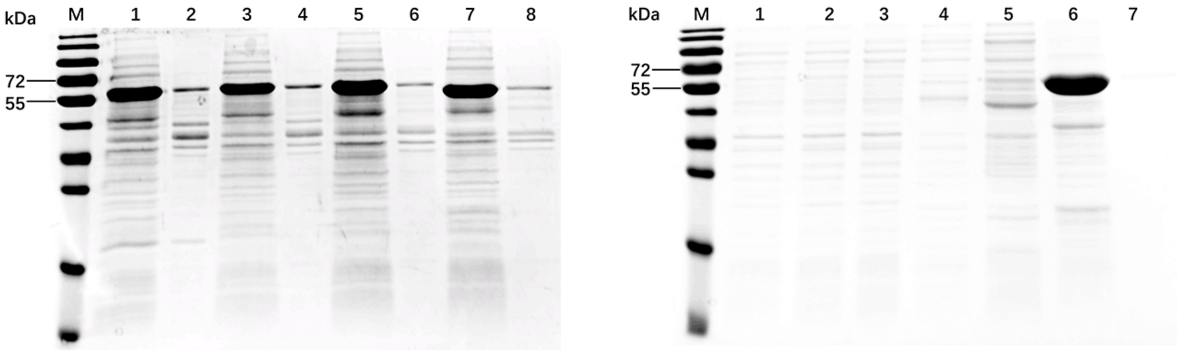

Figure S2. Protein expression purification analysis (a) Lane M- protein marker; Lanes 1, 3, 5, and 7- Bc-HepI, Be-HepI, Bf-HepI, and Bn-HepI recombinant strains of bacteriophage lysate supernatants; Lanes 2, 4, 6, and 8- bacterial lysis precipitate of Bc-HepI, Be-HepI, Bf-HepI, and Bn-HepI recombinant strains; (b) Lane M- protein marker; Lanes 1–7- protein eluate imidazole concentrations of 5, 10, 50, 100, 150, 300, 500. The short black line indicates that the molecular weights of the markers are 72 kDa and 55 kDa.

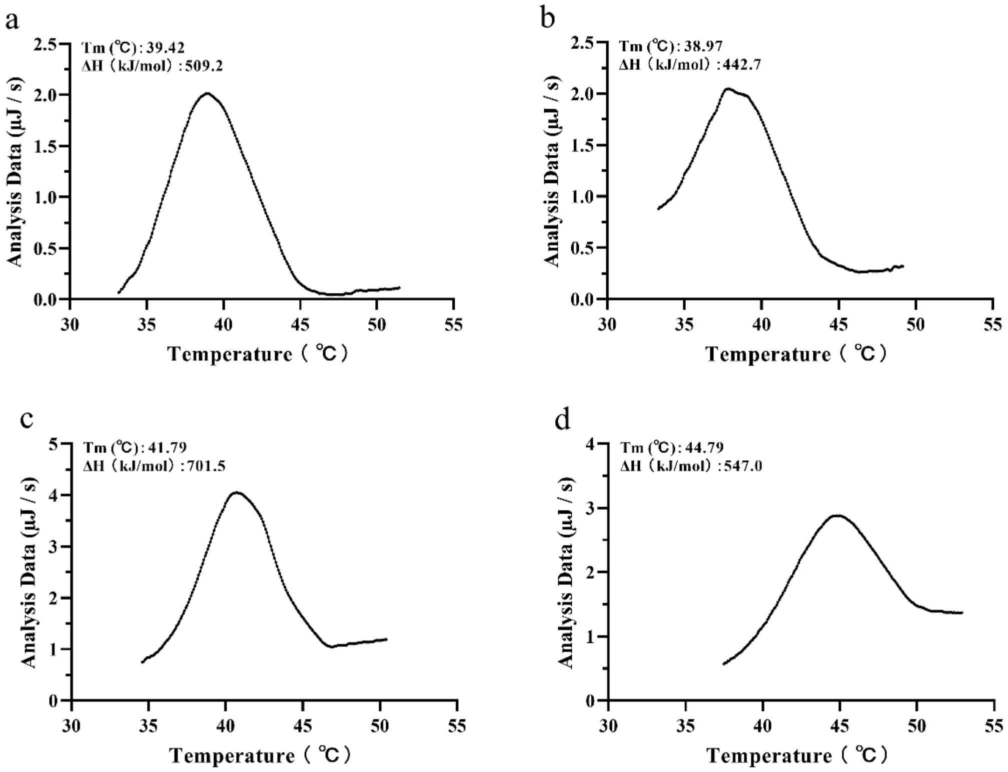

Figure S3. Determination of melting temperatures (*Tm*) of Bc-HepI (a), Be-HepI (b), Bf-HepI (c), and Bn-HepI (d) using differential scanning calorimetry.

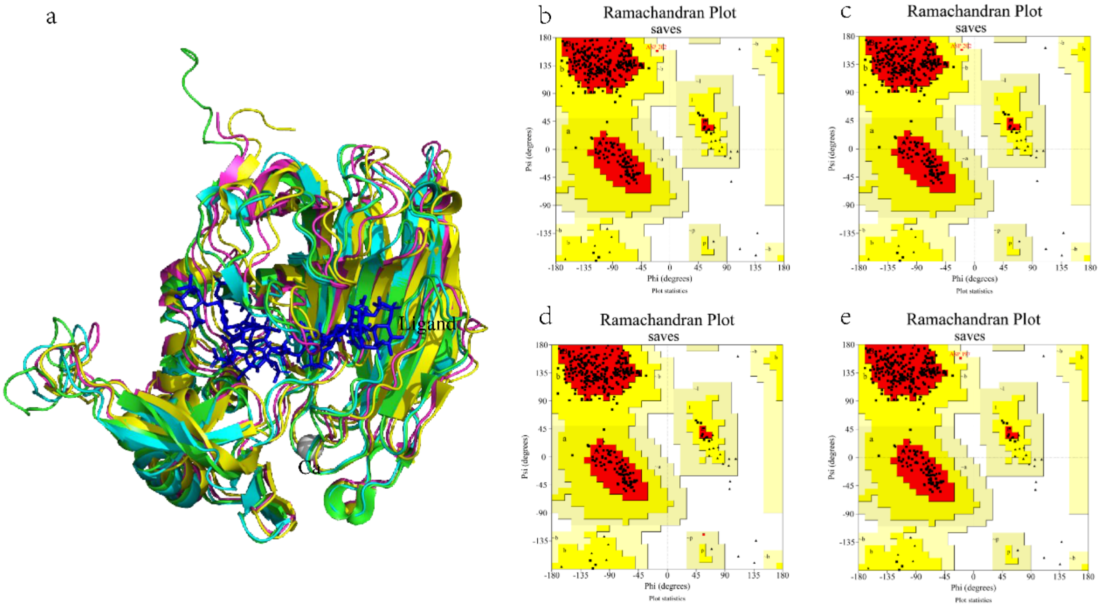

Figure S4. Protein three-dimensional (3D) structure simulation, overlap, and evaluation. (a) The 3D structures of Bc-HepI, Be-HepI, Bf-HepI, and Bn-HepI are shown overlapping. The blue rod-like structure is the substrate ligand. (b-e) Evaluation of the 3D simulated structures of Bc-HepI, Be-HepI, Bf-HepI, and Bn-HepI.
